# REFOCUS Trial: protocol for a cluster randomised controlled trial of a pro-recovery intervention within community based mental health teams

**DOI:** 10.1186/1471-244X-11-185

**Published:** 2011-11-23

**Authors:** Mike Slade, Victoria Bird, Clair Le Boutillier, Julie Williams, Paul McCrone, Mary Leamy

**Affiliations:** 1 King's College London, Health Service and Population Research Department, Institute of Psychiatry, Denmark Hill, London, SE5 8AF, UK

## Abstract

**Background:**

There is a consensus about the importance of 'recovery' in mental health services, but the evidence base is limited.

**Methods/Design:**

A two centre, cluster randomised controlled trial. Participants are community-based mental health teams, and service users aged 18-65 years with a primary clinical diagnosis of psychosis. In relation to the REFOCUS Manual researchintorecovery.com/refocus, which describes a 12-month, pro-recovery intervention based on the REFOCUS Model, the objectives are: (1) To establish the effectiveness of the intervention described in the REFOCUS Manual; (2) To validate the REFOCUS Model; (3) To establish and optimise trial parameters for the REFOCUS Manual; and (4) To understand the relationship between clinical outcomes and recovery outcomes. The hypothesis for the main study is that service users in the intervention arm will experience significantly greater increases in measures of personal recovery (as measured by the QPR) compared to service users receiving care from control teams. The hypothesis for the secondary study is that black service users in the intervention arm will experience significantly greater increases in measures of personal recovery (as measured by the QPR) and client satisfaction (as measured by the CSQ) compared to Black service users receiving care from control teams.

The intervention comprises treatment as usual plus two components: recovery-promoting relationships and working practices. The control condition is treatment as usual. The primary outcme is the Process of Recovery Questionnaire (QPR). Secondary outcomes are satisfaction, Goal setting - Personal Primary Outcome, hope, well-being, empowerment, and quality of life. Primary outcomes for the secondary study will be QPR and satisfaction. Cost data will be estimated, and clinical outcomes will also be reported (symptomatology, need, social disability, functioning).

29 teams (15 intervention and 14 control) will be randomised. Within each team, 15 services users will be randomly chosen, giving a total sample of 435 service users (225 in intervention and 210 in control). Power for the main study: 336 service users will give power to detect a medium effect size of 0.4 (alpha 0.05, power = 0.8) on both QPR sub-scales. Power for the secondary study: 89 participants will give power to detect an effect size of 0.67 on both QPR sub-scales and on CSQ. A range of approaches are used to minimise bias, although service users and clinicians cannot be blinded.

**Discussion:**

This cluster-RCT will evaluate a pro-recovery intervention in community mental health teams.

**Trial registration:**

ISRCTN: ISRCTN02507940

## 1. Background

There is a policy and professional consensus about the importance of 'recovery' in mental health services, defined as "a way of living a satisfying, hopeful, and contributing life" even with any limitations caused by illness [1-4]. This has recently been elaborated: "Recovery is the process of regaining active control over one's life. This may involve discovering (or rediscovering) a positive sense of self, accepting and coping with the reality of any ongoing distress or disability, finding meaning in one's experience, resolving personal, social or relationship issues that may contribute to one's mental health difficulties, taking on satisfying and meaningful social roles and calling on formal and/or informal systems of support as needed" [5].

**Figure 1:**
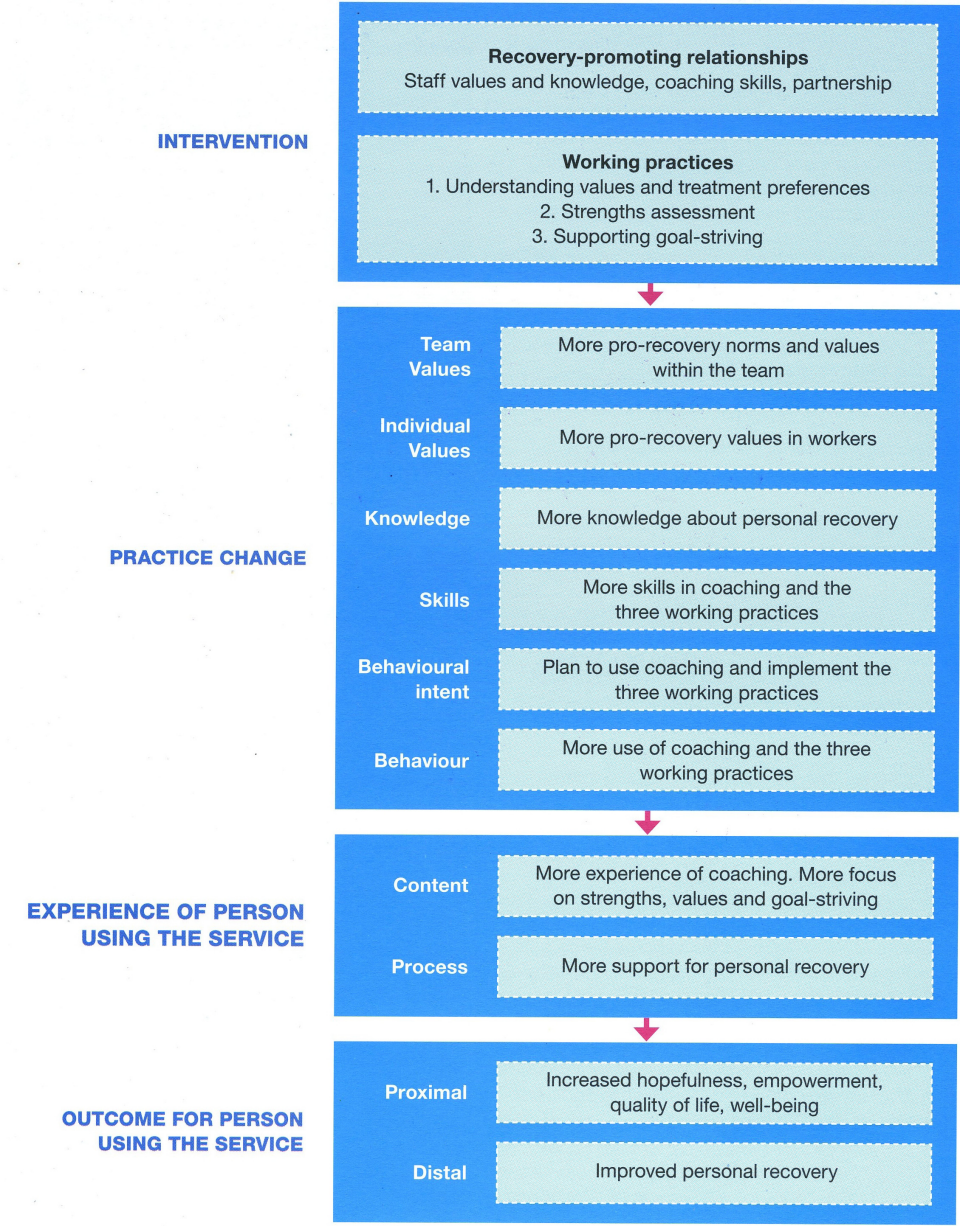
**The REFOCUS Model**.

The REFOCUS Study is a research programme funded through the National Institute for Health Research Programme Grants for Applied Research scheme (RP-PG-0707-10040). The REFOCUS Trial is part of the REFOCUS Study. The REFOCUS Trial is evaluating an intervention (described in the REFOCUS manual [6]) based on the REFOCUS Model, which is derived from wider research [7] and specifically informed by a systematic review and narrative synthesis of personal recovery [8]. The REFOCUS Model is shown in Figure 1.

There is a robust evidence base, including several systematic reviews, for the key elements of the intervention, including the contribution of coaching [9,10], values [11,12], strengths [13,14] and goal-striving [15,16]. This is the first intervention to evaluate their combined use in a complex intervention in adult community mental health services in the NHS in England. The intervention is based on systematic reviews [8] and international best practice [17], and informed by research into staff-service user relationships [18,19] and stigma [20]. Evaluation of the intervention is particularly timely given the current emphasis on recovery and the associated concept of well-being [21] in English mental health services. The results of the trial will be of relevance to (a) developing mental health policy and associated clinical guidelines; (b) clinical practice.

The experiences of recovery of individuals from minority ethnic backgrounds have been insufficiently researched, so a secondary study is being conducted to examine trial outcomes for participants from black backgrounds.

Understanding the relationship between clinical and recovery outcomes is vital if debates about the future direction of mental health services are to be informed by evidence. These debates are happening [22], and the empirical evidence base is very limited [23]. This study will address this knowledge gap.

## 2. Methods/design

### Objectives and hypotheses

#### The REFOCUS Trial has four objectives

**Objective 1: To establish the effectiveness of the intervention described in the REFOCUS manual**, using outcome evaluation to demonstrate that service users receiving care from intervention teams make more progress towards their personal recovery than those receiving care from control teams. The primary outcome measure (QPR) and the secondary outcome measures are listed in Section 13. All outcomes pertain to the individual level and include both staff and service user outcomes.

Main study hypothesis: Service users in the intervention arm will experience significantly greater increases in measures of personal recovery (as measured by the QPR) compared to service users receiving care from control teams.

Secondary study hypothesis: Black service users in the intervention arm will experience significantly greater increases in measures of personal recovery (as measured by the QPR) and client satisfaction (as measured by the CSQ) compared to Black service users receiving care from control teams.

**Objective 2: To validate the REFOCUS Model**, using process evaluation to investigate the extent to which the intended consequences of the intervention are predicted by the REFOCUS Model.

**Objective 3: To establish and optimise trial parameters for the REFOCUS Manual**, including recruitment and retention issues, fidelity, outcome and economic evaluation, implementation strategies, missing data analysis, and sample size calculation

**Objective 4: To understand the relationship between clinical outcomes and recovery outcomes **comprising recovery outcomes of hope, empowerment, well-being, quality of life and personal recovery, and clinical outcomes of symptomatology, needs and social disability.

### Design

This is a two centre cluster-randomised controlled trial, with paired teams randomised to receive the intervention or standard care/treatment as usual arms of the trial. The recovery intervention will be delivered by all members of staff who provide a clinical input to the team.

The intervention will be provided to a complete team, using implementation strategies to support individual practitioners to introduce and maintain these changes. To minimise contamination, the unit of randomisation and analysis is the mental health team. To understand the impact of the intervention a process evaluation will be undertaken.

### Ethics and trial registration

Ethical approval was obtained from East London REC 3 approval 11/LO/0083 on 22.2.11. The trial registration number is ISRCTN02507940 http://www.controlled-trials.com/isrctn.

### Study setting

The intervention will be evaluated in two Mental Health Trusts: South London and Maudsley NHS Foundation Trust (SLaM) and^2^gether Partnership NHS Foundation Trust in Gloucestershire.

SLaM is the largest mental health Trust in the UK, has an annual income of £330 m, spent across over 100 sites spanning urban and suburban settings. It employs 4,500 staff in 296 teams, works with 34,128 service users, and provide adult mental health services across four Boroughs (Croydon, Lambeth, Lewisham, Southwark). These services are provided through Clinical Academic Groups (CAGs). CAGs bring together clinical services, research, education and training for the benefit of patient care. People who use SLaM services are ethnically diverse, with 37% of people using SLaM services recorded on the clinical information system as coming from a 'Black African', 'Black Caribbean' or 'Black other' background.

^2^gether is a rural/semi-rural Trust, employing 806 staff in 23 adult mental health teams, and working with 4,301 service users. People who use^2^gether services are ethnically homogenous, with a very small number of black individuals using services. Therefore the secondary study will be conducted in SLaM only.

### Sample

Team inclusion criteria

• Adult, community-based mental health team (due to the diversity in in-patient provision [24,25])

• Any Complex Care or Promoting Recovery team in the SLaM Psychosis Clinical Academic Group (CAG) or any in^2^gether

• Provide a care co-ordinating function

Service user inclusion criteria

• Aged 18-65 years

• Primary clinical diagnosis of psychosis, e.g. schizophrenia, schizo-affective disorder, bipolar disorder

• No immediate plans for discharge or transfer

• Not currently receiving in-patient care or in prison

• Speaks and understands English

• Not participating in substantial other study

• Has participating paired staff

• In opinion of clinician, is sufficiently well to participate

• In regular contact with at least one worker in the team

Service user exclusion criteria

• Service users who are unable to give consent or are too unwell to be interviewed (in the opinion of clinician)

• No participating paired staff

• Service user whereabouts unknown or service user is uncontactable

Additional service user inclusion criteria for secondary study

• From black African, black Caribbean, black British or black other backgrounds

Staff inclusion criteria

• Provides clinical input into a team included in the trial

• Does not also provide clinical input into a team allocated to the opposite arm of the trial.

• In addition, paired staff completing staff-rated service user measures are in regular clinical contact with service users who are recruited into the trial.

### Sample size

#### Main study

The primary outcome is the Process of Recovery Questionnaire (QPR) [26]. This measure was chosen as the only service user-rated measure of personal recovery which has been developed in England and with adequate psychometric properties (described in Section 13.2). Since personal recovery is something experienced rather than assessed by an expert, a self-report measure was appropriate for clinical end-point. The timing between baseline and follow-up (*i.e*. the length of the intervention) was chosen as 12 months to allow sufficient time for team-level changes in practice to occur, be sustained, and have an impact.

The QPR has two sub-scales: intrapersonal (mean = 45.7, sd = 16.1, range 13-68) and interpersonal (mean = 14.0, sd = 3.7, 0-20). The sample size calculation is based on being able to detect a medium standardised effect size of 0.4 in both sub-scales, which equates to a difference of 6.4 points on the QPR intrapersonal subscale and 1.5 points on the interpersonal subscale.

The estimated sample size for a two-group comparison of means (alpha = 0.05, power = 0.8) is 99 per group. However, as this is a team-level intervention, the unit of randomisation is the team. An initial 29 teams (20 from SLaM, 9 from^2^gether) will be recruited, and we anticipate 17% attrition (due to team mergers or restructuring), giving a total of 24 teams (16 from SLaM, 8 from^2^gether). The same number of participants will be included from each team. Adjustment for clustering within teams assumes an intracluster correlation of 0.05, with equal numbers of clusters in each randomisation group. For 24 teams, 164 participants are needed per group, i.e. 14 participants per team. To allow for one participant (7%) drop-out per team, 15 participants per team will be recruited from the 29 teams. This drop-out rate is consistent with attrition in previous randomised controlled trials we have conducted [27].

The total initial sample is therefore 435 service users, comprising 225 in intervention and 210 in control. Based on the above attrition and clustering estimates, we anticipate this will produce an analysable sample of 336 participants, giving power to detect a medium effect size of 0.4 (alpha 0.05, power = 0.8) on both QPR sub-scales.

#### Secondary study

In a pre-planned sub-group analysis, we will also be investigating the outcomes of service users from black backgrounds in the SLAM site only. The primary outcomes for this sub-study will be the QPR (as above) and the Client Satisfaction Questionnaire - 8 item version (CSQ) [28], which produces a global satisfaction score (mean = 24, sd = 6) within this population. CSQ data were available from a previous study [29], from which a retrospective analysis of data from black individuals demonstrated an effect size of 0.67 for differences in CSQ between intervention and control groups. The sample size calculation for the secondary study is based on being able to detect a large effect size of 0.67, equating to a difference of 10.8 on the QPR intrapersonal subscale, 2.5 on the QPR interpersonal subscale, and 4 on the CSQ.

Using the same estimates for a two-group comparison of means as for the main study (20% SLaM team attrition, 7% participant attrition, 0.05 intra-cluster correlation, alpha 0.05, power 0.8), 6 participants per team will be recruited. The initial sample of 120 (20 teams × 6 participants) is anticipated to produce an analysable sample of 89 participants, giving power to detect an effect size of 0.67 on both QPR sub-scales and on CSQ.

### Recruitment and randomisation procedures

Teams will be recruited into the trial over a 12 month period, at a rate of 5-6 SLaM teams every three months and 4-5^2^gether teams every five months. One of the aims of this trial is to establish whether this planned recruitment rate is feasible. The service user baseline assessment measures are anticipated to take up to 90 minutes, so if necessary will be completed in two face to face meetings with researchers. The generic staff baseline assessment will take up to 20 minutes and the paired staff baseline assessment measures will take an additional 20 minutes per service user. They will either be completed at the community base or within the service user's own home, at a mutually agreed time.

Participating teams will be randomly allocated on an equal basis to intervention (N = 15) and control groups (N = 14). Allocation will be stratified by site (six sites: four SLaM Boroughs, two^2^gether localities) to ensure balance. Randomisation will be undertaken using processes set out by the independent Mental Health and Neuroscience Clinical Trials Unit, on the basis of a team identification number and site information. Service users will be allocated based on these clusters.

The clinical information system will be accessed by either Clinical Studies Officers (CSOs) from National Institute for Health Research (NIHR) Mental Health Research Network (MHRN), information analysts or the researchers acting under arrangements with the responsible Trust to compile the list of names, diagnoses, ethnicity and date of births for randomisation. This will be the only access to clinical information by the research team prior to consent.

In^2^gether, a randomly ordered list of service users on the caseload of each team with a clinical diagnosis of psychosis will be generated using the same procedures as set out by the Clinical Trials Unit. The first 15 service users will be selected from the randomly ordered caseload list. In SLaM, two randomly ordered lists of service users with a psychosis diagnosis on the caseload of the team will be generated using a random number table. One list (List A) will comprise service users who come from service users who are from black African, black Caribbean and black other backgrounds, and the other list (List B) will comprise all other service users. The first 6 service users from list A and the first 9 from list B will be selected, giving a total sample of 15 per SLaM team. This will ensure epidemiological representativeness in the sample in relation to black ethnicity, and will ensure sufficient power to test the secondary study hypothesis.

If an individual does not meet inclusion criteria or refuses consent to participate, then the next person from the appropriate randomly-ordered list will be chosen. Caseload randomisation will be undertaken using procedures set out by the Mental Health and Neuroscience Clinical Trials Unit, on the basis of a service user identification number.

### Approaches to minimise bias

Due to the nature of the intervention, neither clinicians nor service users can be blinded to allocation status. However, several approaches have been used to minimise bias.

Addressing bias at allocation

1. All randomisation will be undertaken by the research team following procedures set out by the independent Clinical Trials Unit which has been awarded full CTU registration by UKCRC. Identifying information about teams or service users will not be known before randomisation.

Addressing bias in baseline data

2. Baseline data from staff and service users will as far as feasible be collected before allocation, to avoid bias based on allocation status

Addressing bias in the intervention

3. The research team will monitor implementation across sites, in order to maximise fidelity and ensure comparability of the intervention between sites.

4. Change in staff for each team will be carefully monitored, with particular attention paid to identifying staff that move between teams in different allocation arms. This will allow contamination to be estimated.

Addressing bias in follow-up data

5. All included services users will be followed up and included in the analysis using intention-to-treat approaches, reducing the impact of selective attrition.

6. At follow-up assessment, participants will be asked not to reveal their allocation status, and at the end of the interview the rater will record their guess about the service user's allocation status. This will allow researcher blindness to be estimated.

7. Follow-up data will where possible be collected by CSOs from the National Institute for Health Research Mental Health Research Network (MHRN). This will increase the likelihood of rater blindness.

8. Bias in the outcome data will be minimised by the use of standardised objective assessments, and informed by previous outcomes research [30,31].

9. Some evaluation data will be obtained from casenotes, thus reducing the possibility of respondent bias. More generally, the multi-method approach to evaluation includes data collected from staff, service users, researcher ratings, and case note audit. This reduces the impact of bias in any individual data source.

10. The focus group process evaluation data, for which unblinding will be necessary as only intervention group participants are involved, will be collected after the follow-up outcome evaluation data.

11. Protected data storage with clear access protocols in line with Good Clinical Practice Guidelines [32] will be used to store allocation and outcome data separately.

Addressing bias in analysis

12. No data regarding the allocation status will be stored in the data entry database

13. All data will be stored in either a locked filing cabinet or in an electronic password-protected database.

14. The primary data analysis will be undertaken blind to allocation status

15. Allocation status will coded as A and B in all data requests.

### Control

All participating teams provide care co-ordination for clients. The framework for care co-ordination and resource allocation in mental health care is the Care Programme Approach (CPA) [33]. The CPA process is well-established in the trial sites. Key components of this approach include:

• Systematic arrangements for assessing the health and social needs of people accepted into specialist mental health services

• The formation of a care plan which identifies the health and social care required from a variety of providers

• The appointment of a key worker to keep in close touch with the service user and to monitor and co-ordinate care

• Regular review and, where necessary, agreed changes to the care plan.

• Individuals will continue to receive treatment as usual, directed by the principles and CPA process outlined above.

Individuals within the control teams will continue to receive treatment as usual, as directed by the principles and CPA process outlined above.

### Intervention

The intervention comprises a 12-month, team-level pro-recovery intervention in addition to standard care. It includes recovery interventions to be carried out at both the team (cluster) and individual practitioner level. The intervention is described in detail in the REFOCUS manual (available from http://researchintorecovery.com/refocus). In summary, it involves two components:

#### A. Recovery-promoting relationships

• Training and reflection opportunities will be offered to teams to allow them to understand what personal recovery means in their context, to consider their own values and how these can support recovery, and to develop and practice the use of coaching skills.

• People who use services can be active agents in shaping the content of clinical interactions. Service users will be supported to develop expectations that their values, strengths and goal-striving will be prioritised.

• Partnership relationships recognise the professional expertise of staff and the expertise from lived experience of service users. Teams will undertake a project to develop and practise partnership working, e.g. through staff and service users doing or learning something jointly.

#### B. Pro-recovery working practices

• Understanding the service user's values and treatment preferences underpin an individualised approach to care planning. Workers will be trained in understanding values.

• Amplifying a service users' strengths and ability to acccess community supports is an important approach to supporting recovery. Workers will be trained in assessing strengths.

• Identifying personally valued goals, developing intermediate steps, and striving towards these goals contributes to recovery. Workers will be trained to use existing care planning skills to support goal-striving.

The practice change is supported by an implementation strategy, described in detail in the REFOCUS manual. In summary, the process involves six implementation components: i) Information sharing; ii) Personal recovery training; iii) Coaching and working practice training; iv) Team manager reflection groups; v) Team reflection sessions; and vi) Supervision reflection.

### Measures

#### Staff-Rated Measures

##### Generic

• The **Sociodemographics Form - Worker (SF-W) **is a staff-rated form developed specifically for the trial, which records the person's age, gender, ethnicity, languages spoken, qualifications, core profession, current role, grade, team, time since qualification, personal experience of mental illness, and experience of caring for a family member or friend with a mental illness.

• The **Recovery Knowledge Inventory (RKI) **is a 20-item staff-rated measure of staff knowledge and attitudes about recovery [34].

• The **Mental Illness: Clinicians' Attitudes (MICA) **Scale is a 16-item of attitudes towards mental illness (staff-rated) [35].

• The **Recovery Fidelity Scale - Worker (RFS-W) **is a staff-rated measure developed specifically for the trial, which assesses fidelity to the intervention elements.

• The **Implementation Scale (RIS) **is a staff-rated measure developed specifically for the trial, which assesses the experience of the six implementation strategies.

##### Service user-specific

• The **Health of the Nation Outcome Scale (HoNOS) **is a 12-item staff-rated measure of social disability [36].

• **Camberwell Assessment of Needs Short Appraisal Schedule-(CANSAS - S) **is a 22-item staff-rated assessment of health and social needs [37].

• The **Global Assessment of Functioning (GAF) **is a 3-item staff rated measure of impairment in functioning due to physical (or environmental) limitations.

#### Service User-Rated Measures

##### Outcome measures

• (Primary outcome) The **Process of Recovery Questionnaire (QPR) **is a 22-item service user-rated assessment of personal recovery [26]. Each item is rated on a five-point scale from 0 (Disagree Strongly) to 4 (Agree strongly). Two subscale scores are produced: Intrapersonal sub-scale based on 17 items (range 0 to 4, high score good) and the Interpersonal sub-scale based on 5 items (range 0 to 4, high score good). The QPR was developed in the UK and has been used with a population of people who have experienced psychosis. It has an internal consistency of r = 0.94 for the Intrapersonal sub-scale and r = 0.77 for the Interpersonal sub-scale, indicating good internal consistency. It obtained good test-re-test reliability for both sub-scales (Intrapersonal: r = 0.874, p = 0.001) and (Interpersonal: r = 0.769, p = 0.001). It also has good construct validity and correlates with the Making Decisions Empowerment Scale, Schizophrenia Quality of Life Scale and General Health Questionnaire.

• (Secondary outcome) The **Warwick-Edinburgh Mental Well-Being Scale (WEMWBS) **is a 14-item service user-rated of well-being [38].

• (Secondary outcome) The **Mental Health Confidence Scale (MHCS) **is a 16-item service user-rated measure of empowerment [39].

• (Secondary outcome) The **Herth Hope Index (HHI) **is a 12-item service user-rated measure of client levels of hope [40].

• (Secondary outcome) The **Manchester Short Assessment of Quality of Life **(**MANSA) **is a 16-item service-user rated measure of quality of life [41].

• (Secondary outcome) The **Goal setting - Personal Primary Outcome (GS-PPO) **is a service user-rated measure developed specifically for this trial, which identifies a personally-valued outcome as an alternative to the pre-defined primary outcome of the trial.

• (Secondary outcome) **Client Satisfaction Questionnaire (CSQ) **is an 8-item service user-rated measure of client satisfaction with mental health services [28].

• **Camberwell Assessment of Needs Short Appraisal Schedule-(CANSAS - SU) **is a service user-rated 22-items measure of health and social needs [37]. Both staff and service user perspectives are assessed becase they differ [42,43]

• **ICECAP-A **is service user-rated 5-item measure of overall quality of life which intended for use in economic evaluation [44].

##### Process measures

1. The **Importance of services in recovery (INSPIRE) **is a service user-rated measure of recovery orientation of services which was developed specifically for this trial.

2. The **Recovery Fidelity Scale - Service User (RFS-SU) **is a service user-rated measure developed specifically for this trial, which assesses experience of the intervention elements. Each item is rated on a five-point scale ranging from 0 (Strongly Disagree) to 4 (Strongly Agree). The Total score is calculated by summing each item and multiplying by 25. The total score ranges from 0 (low fidelity) to 100 (high fidelity).

##### Other measures

3. The **Sociodemographics Form - Service User (SF-SU) **is a service user-rated form developed specifically for the trial, which allows recording of the service user's date of birth, gender, ethnicity, languages spoken, country of birth, education, employment, marital status, and housing. It also records (in tear-off form for separate filing) a range of contact details (e.g. mobile, email, contact information for a relative) to facilitate contact with service users who are no longer on the caseload of the team at follow-up.

10. The **National Adult Reading Test (NART) **is a measure of pre-morbid level of intellectual functioning [45].

#### Researcher-Rated Measures

1. The **Brief Psychiatric Rating Scale (BPRS) **is an 18-item observer-rated measure of symptomatology which is completed with the service user [46].

2. The **Client Service Receipt Inventory (CSRI) **is a tool for collecting cost-related information about people with mental health problems for use in mental health service evaluations [47].

#### Team-Manager/leader measures

1. The **Team Characteristics Form (TC-F) **is a team manager/leader-rated form developed specifically for the trial, which records the team's key characteristics, such as caseload size, composition, type of team and support offered, and whether it has undergone any significant changes within the last 12 months.

### Trial procedures

Eligible teams will be recruited into the trial over a 12 month period, starting April 2011. Baseline data collection will start in teams four to five months prior to the start of the intervention. All staff who meet inclusion criteria in the participating teams will be asked to complete the generic staff measures: SF-W, MICA, RFS-W and RKI. Staff will identify whether each randomly identified service user meets the eligibility criteria. Eligible service users will then be approached by their Care Co-ordinator or another appropriate staff member from their team and asked whether they are willing to be contacted by researchers. If they agree to be contacted, then Clinical Studies Officers or research workers will then approach the potential participants, in liaison with staff. Potential participants will be given a detailed explanation of the study and a written Participant Information Sheet. If they are willing to participate, they will be asked to sign a consent form and complete the baseline interview, comprising: QPR, WEMBWS, MANSA, GS-PPO, CSQ, RFS-SU, INSPIRE-TEAM, NART, MHCS, HHI, CANSAS-SU, ICECAP-A, SF-SU, CSRI, and BPRS. If necessary, the baseline assessment will be spread over more than one meeting. Participants will be paid £10 for taking part and entered into a raffle competition to win a £100 gift voucher.

Following baseline assessment of the service user, their care co-ordinator or other appropriate team member will be asked to complete the three service user-specific measures: HoNOS, CANSAS-S and GAF.

Four to five months (varying across sites) after baseline data collection began, teams will be randomly allocated with 50% likelihood into either the intervention or control groups. The intention is that all baseline data will be collected before allocation, but where this is not possible any remaining baseline data will be collected post-allocation. On completion of the baseline assessments, teams will be given a £100 book voucher.

The 12-month intervention will be provided for intervention group teams only. Attendance at all training and reflection sessions will be recorded.

At the intervention mid-point (6 months) team leaders in the intervention teams will be asked to complete the RFS-W.

At the end of the intervention, all service users who completed baseline assessments will be re-contacted in liaison with their care co-ordinator. Where the service user is no longer in contact with the team, efforts will still be made to contact them for a follow-up assessment. The follow-up assessment will be conducted by the CSO or research worker, and will comprise the following measures: QPR, WEMBWS, MANSA, G-S-PPO, CSQ, RFS-SU, INSPIRE-TEAM, MHCS, HHI, CANSAS-SU, ICECAP-A, SF-SU, CSRI, and BPRS. The interviewer will guess allocation status at the end of the interview. If necessary, the follow-up assessment will be spread over more than one meeting. Participants will be paid £10 for taking part and entered into a raffle competition to win a £100 gift voucher.

After the follow-up assessment of the service user, their care co-ordinator or other appropriate team member will be asked to complete the three service user-specific measures: HoNOS, CANSAS-S and GAF. They will also identify the current primary clinical diagnosis. If the baseline staff respondent is no longer working with the service user, then the most appropriate member of staff will be asked to complete the measures instead.

All staff in intervention group teams (whether paired with service users or not) will be asked to rate RKI, MICA, RFS-W and RIS. All staff in control group teams will be asked to rate RKI, MICA and RFS-W. Any new staff in either group will also complete SF-W. The trial is planned to start in April 2011, with follow-up completed by December 2013.

A summary of the assessment measures is shown in Table 1.

**Table 1:** Assessment measures in the REFOCUS Trial

Measure	Completion time (mins)	Time		
*Team managers*				

TC-F	2	B		FU

*All staff*				
SF-W	3	B		FU*
RKI	5	B		FU
MICA	5	B		FU
RFS-W	5	B	MP	FU
RIS (intervention staff only)	3			FU

*Paired staff - completed for each service user*				
HoNOS	10	B		FU
CANSAS-S	10	B		FU
GAF	3	B		FU

*Service user*				
SF_SU	2	B		FU
NART	10	B		
QPR	10	B		FU
WEMWBS	5	B		FU
MHCS	5	B		FU
HHI	5	B		FU
MANSA	10	B		FU
GS-PPO	10	B		FU
CSQ	5	B		FU
CANSAS-SU	10	B		FU
INSPIRE	10	B		FU
RFS_SU	3	B		FU
ICECAP-A	5	B		FU

*Researcher rated*				
BPRS	10	B		FU
CSRI	10	B		FU

Key:

B = Baseline

MP = Mid point 9 months after baseline, completed by team manager in intervention teams

FU = Follow up at 12 months post-baseline

* for new staff only

### Process evaluation

In addition to the baseline and follow-up data collected, four process evaluation approaches will be used.

#### 1. Longitudinal routine data

Longitudinal routine data will be collected from the clinical information system at six-monthly intervals starting one year before baseline and continuing until follow-up (i.e. at baseline - 12 months, baseline - 6 months, baseline, baseline + 6 months, baseline + 12 months, baseline + 18 months). At each recording point, anonymised data will be collected from the care plans for the participating service users. Each care plan entry will be rated for Topic and Responsibility, using categories developed in a previous study [48].

Topic categories comprise: Psychotropic medication; Symptoms/Relapse prevention; Care Programme Approach; Outpatient clinic; Physical health; Social needs; Emotional Support; Functional/Activities of Daily Living; Accommodation needs; Healthy Lifestyle; Financial; Employment; Education; and Carer work/Support.

Responsibility categories identify who is responsible for actions in the care plan, and comprise: Staff; Service user; Staff and service user; Carer; Staff and Carer; Service user and Carer; and Staff, service user and carer. Data will be collected for each consenting service user participant.

Anonymised data will be collected from the three clinical information system fields: Values and treatment preference; Strengths; and Personally valued goals.

#### 2. Interviews

Staff participants in individual interviews and focus groups will comprise intervention group (a) front-line community mental health team staff that have direct clinical contact with service users (b) team leaders/managers. Service user participants in individual interviews and focus groups will comprise people on the caseload of intervention group teams. Trainer participants will comprise (a) Personal Recovery trainers and (b) Coaching for recovery trainers.

##### Interviews with staff (N = 30)

Interviews will be held midway through the trial (6 months), with individual members of staff from the intervention teams at both sites (N = 15). The topic guide will explore the experience of participating in the trial, and identify implementation influences.

At the end of the trial (12 months), interviews will be held with individual members of staff from the intervention teams, about their experience of taking part in the study and in what ways (if any) their working practices have changed (N = 15). The topic guide will also cover their views on incorporating recovery interventions within their routine clinical practice and the impact on service users of implementing the intervention.

##### Interviews with service users (N = 30)

Interviews will be held midway through the trial (6 months) with service users from the intervention teams at both sites (N = 15). The sample will include at least five SLaM service users from black African, black Caribbean and black Other backgrounds. At the end of the trial (12 months), 15 participating service users (including at least five SLaM service users from black African, black Caribbean and black Other backgrounds) from intervention teams will be interviewed at follow-up about their experience of taking part in the study, how the interventions were delivered in practice, and what impact the intervention has had on their relationships with clinicians and their recovery more generally.

##### Inerviews with trainers (N = 2)

Interviews will be held with Personal Recovery and Coaching for Recovery trainers, midway through the trial (6 months).

#### 3. Focus Groups

##### Team focus groups (n = 4)

At 12 months, four intervention group teams will be purposively selected which are most successful (n = 2) and least successful (n = 2) at implementing the intervention, based on RFS-W scores. Separate focus groups with the staff from each identified team will be held, using a topic guide to explore the blocks and enablers of implementation, along with the experience of participating in the intervention.

##### Service user focus groups (n = 4)

At 12 months, four focus groups will be held with service users from a high implementation team (n = 1), low implementation team (n = 1) and service users from black African, black Caribbean and black other backgrounds (n = 2). The topic guide will explore the experience of care, and whether any aspects have changed.

All qualitative data (staff and service user focus groups and interviews) will be audio taped and fully transcribed. Transcripts and notes will be read and re-read independently by two of the research team. The data will then be organised into initial codes and higher codes that provide insight into emergent themes. Reliability will be enhanced by identifying issues that are consistent between groups. A computer software package will be used to manage the data and increase the transparency of the analysis. Deviant cases will be actively sought throughout the analysis and emerging ideas and themes modified in response. Interim analysis will identify modifications to the topic guide.

#### 4. Training Evaluation Reports

End of training reports from Personal Recovery and Coaching for Recovery trainers, for each of the intervention teams (N = 30), specifically focussing upon the REFOCUS model, will be analysed qualitatively. In addition, individual participant feedback evaluation forms on each session of both the Personal Recovery and Coaching for Recovery training will be analysed quantitatively.

### Analysis plan

#### Objective 1: To establish the effectiveness of the intervention described in the REFOCUS manual

The two trial hypotheses will be investigated using standard intention-to-treat analysis for all participants with end-of-trial data. Firstly, to identify whether the intervention was associated with improved personal recovery (QPR) and secondly, whether it was associated with improved secondary outcomes at the end of the intervention. Individual level outcomes for staff and service users will be aggregated at the cluster level. Analysis of covariance (ANCOVA) will be used to adjust for baseline levels including clustering effects of teams and other baseline prognostic indicators. Sensitivity analysis will control for baseline covariates associated with missing data. Analysis will be blind to allocation status.

#### Objective 2: To validate the REFOCUS Model

The extent to which the REFOCUS Model accurately captures changes will be tested through process evaluation. The Intervention component will be validated using data from RFS-W and RIS, along with data about attendance at training and reflection sessions. The practice change component will be validated using data from MICA, RKI, RFS-SU, RFS-W, RIS, staff focus groups and interviews, and longitudinal routine data. The service user experience component will be validated using data from RFS-SU, INSPIRE, and service user focus groups and interviews. The Outcome component will be validated using data from the primary outcome QPR and the secondary outcomes (CSQ, GS-PPO, HHI, WEMWBS, MHCS and MANSA). In the light of this analysis, the REFOCUS Model will be modified if necessary. A test of subgroup-treatment effect interaction will be conducted for the *a priori *sub-group analysis of individals from black backgrounds.

#### Objective 3: To establish and optimise trial parameters for the REFOCUS manual

Using the elements identified in the frameworks for optimising trial parameters [49,50], the trial analysis will focus on the following:

### Recruitment and retention

Issues arising during recruitment and factors influencing retention will be noted, and used to inform recommendations for a recruitment and retention strategy in a future trial. Additionally, the recruitment and retention rates will directly inform a future sample size calculation.

### Treatment fidelity

The extent to which the intervention was provided as planned will be investigated using data from RFS-W, RIS, the staff focus groups and semi-structured interviews, and the longitudinal routine data. Contamination will be assessed by comparing data from RFS-W for intervention and control group respondents.

### Implementation

Data from the RIS measure and the staff focus groups and semi-structured interviews will be used to identify implementation components which were either highly effective or highly burdensome. Team-level aggregated data from the service user-level and the generic staff-level measures will be analysed to compare the groups of the trial and, where possible, to track longitudinal changes over time. This will include using interrupted time series approaches with the longitudinal routine data, to detect abrupt changes in the time course of process indicators, and relate them to changes in inputs.

### Missing data

Sensitivity analyses will be performed to assess the influence of lost to follow-up and refusals, including imputation of missing baseline values from the within-site means; multiple imputation of follow up values (where feasible from other variables) and controlling for any baseline variables that are related to missing outcomes in the analysis.

### Sample size calculation

Data from the trial will inform a sample size calculation for a future RCT, including strengthening the robustness of estimates of participant retention, clustering effect, and intervention effectiveness.

### Economic evaluation

The health economic component will consist of an analysis of the cost impact of the intervention and the link between costs and outcomes in the form of a cost-effectiveness analysis. A broad costing perspective will be used for the economic evaluation. A societal perspective will be adopted, thereby capturing the costs of healthcare, social care, informal care from family/friends and also lost employment. The Client Service Receipt Inventory (CSRI) will be used for this purpose. CSRI data will be combined with nationally applicable unit costs from published sources [47]. Cost data are usually skewed, so bootstrapping will be used to generate confidence intervals around the cost differences. Cost-effectiveness (C-E) will be assessed by combining the cost and outcome data. Incremental C-E ratios will indicate the extra cost (if any) incurred to produce an extra unit gain in outcome. Uncertainty in the C-E estimates will be explored using C-E planes and C-E acceptability curves. The main outcome measures for the economic evaluation will be those that are used for the overall evaluation. It will also be possible to measure Quality Adjusted Life Years (QALYs). The feasibility of this approach will be explored further during the analysis stage.

### Evaluation strategy

The comprehensive battery of assessments will allow identification of measures which provide the most useful data for both outcome and process evaluation. Any emergent approach to increasing blindness will be identified.

#### Objective 4: To understand the relationship between clinical and recovery outcomes

The relationship between clinical outcomes (HoNOS, CANSAS-S, CANSAS-SU, BPRS, GAF) and recovery outcomes (QPR, HHI, MHCS) will be investigated using multivariate modelling.

### Trial Management

Dr Mike Slade (PI) will have overall responsibility for the trial. The Trial Manager is Dr Mary Leamy (Health Service and Population Research Department, Institute of Psychiatry), who will be responsible for co-ordination.

### Risk and adverse events

Relevant trust policies relating to potential areas of risk, such as risk management and medication, will be adhered to. Serious adverse events will be monitored by the Trial Manager, who will report these to the PI and where there is a possibility that they are linked to the trial, the Trial Steering Committee will be informed.

### Trial supervision

The **Trial Steering Committee **will comprise four independent members: Prof Sonia Johnson, Department of Mental Health Sciences, University College London (chair); Nora Donaldson, King's College London; Pauline Edwards, University College London; and Caroline Cuppitt, Oxleas Mental Health NHS Trust. In addition, the Principal Investigator (Mike Slade), the site lead (Rob MacPherson) and the Trial Manager (Mary Leamy) will attend. At the first TSC meeting the need for a **Data Monitoring and Ethics Committee**, interim analyses and stop rules for the trial will be discussed and a Data Monitoring and Ethics Committee will be convened if necessary.

The REFOCUS trial is part of the Refocus Study, which is supported by four advisory committees [51]. The **Steering group **comprises co-applicants and invited experts. The members are: Gyles Glover, Gabrielle Richards, Geoff Shepherd, Vanessa Pinfold, Jerry Tew, Shula Ramon, Tom Craig, Zoe Reed, Lynne Turner-Stokes, Rachel Churchill, Graham Thornicroft, Rachel Perkins, John Larsen, Rob Macpherson, Joanna Fox, John Weinman, Guy Saward, Morven Leese, Paul McCrone, Tony Coggins and John Larsen. The **International Advisory Board (IAB) **comprises: Glenn Roberts, Mark Hayward, Michael Clark, Simon Bradstreet, Tom O'Brien, Larry Davidson, Lindsay Oades, Marianne Farkas and Courtenay Harding. The **Lived Experience Advisory Panel (LEAP) **comprises 8 people who have experience of either using mental health services or caring for someone who has. The **Black and Minority Ethnic Virtual Consultation Panel **is made up of 12 members including researchers, clinicians and service users either from black backgrounds or with extensive experience of working with individuals from black backgrounds. These have been meeting since 2009, and will continue to meet during the trial.

### Data Handling and Record Keeping

The two trial sites will enter data independently into secure password-controlled databases held locally. Data entry will include validation checks. Data will periodically be merged to allow interim analysis, as requested by the TSC and as needed by the Trial Manager to monitor progress. The only documentation which will contain identifying material are the participant contact details (tear-off portion of the SF-SU) and consent forms (containing name). Any audiotape recordings will be destroyed once the transcription has been checked for accuracy. All paper forms of this data will be stored in locked filing cabinets at each of the sites and transferred to the Institute of Psychiatry at the end of the study. Only the research team will have access to these filing cabinets. All other data will be identified by a Participant Identification Number only. A file linking the Participant Identification number and personal data will be password protected and stored on a secure server at the Institute of Psychiatry. Only the research team will have access to this data. Electronic and paper data will be retained for 10 years. All members of the study team will receive MRC Good Clinical Practice training in RCTs and we shall follow Research Governance arrangements.

### Data Access

This study will generate qualitative data comprising interview transcripts and associated analyses, and quantitative data from questionnaires. Exclusive use for primary research by the research team is envisaged for no more than 3 years following the study, to meet dissemination goals. Both the quantitative and qualitative data will be shared in anonymised form. It is anticipated that the data may be used for secondary re-analysis as well as contributing to larger datasets of routinely collected outcome data. Archiving and curating (including data sharing agreements and management of access rights) will be undertaken within the framework used by King's College London, with due attention to issues of ethical (including consent and confidentiality aspects), legal and institutional regulatory permissions. Intellectual property rights for the data will be retained by the lead applicant and King's College London.

### Publication

The results of the research will be targeted for publication in peer-reviewed journals of general and special interest.

## Competing interests

The authors declare that they have no competing interests.

## Authors' contributions

MS obtained the funding, led the study design and drafted the manuscript. All authors contributed to the design, and read, contributed to and approved the final manuscript.

## Authors information

Further information about this research group is at http://researchintorecovery.com.

## Pre-publication history

The pre-publication history for this paper can be accessed here:

http://www.biomedcentral.com/1471-244X/11/185/prepub
